# Efficacy and Safety of Endoscopic Resection for Small Gastric Gastrointestinal Stromal Tumors in Elderly Patients

**DOI:** 10.1155/2022/8415913

**Published:** 2022-04-23

**Authors:** Changzhou Cai, Jinpu Yang, Mengting Ren, Lu Lv, Xinxin Zhou, Mosang Yu, Feng Ji

**Affiliations:** Department of Gastroenterology, The First Affiliated Hospital, Zhejiang University School of Medicine, #79 Qingchun Road, Hangzhou, Zhejiang Province, China 310003

## Abstract

**Background:**

Gastrointestinal stromal tumors (GISTs) are prevalent in elderly patients. Endoscopic resection has become popular for treating small (≤5 cm) gastric GISTs. However, little is known about the outcomes of endoscopic resection in elderly patients.

**Aim:**

To assess the efficacy and safety of endoscopic resection for small (≤5 cm) gastric GISTs in elderly patients (≥65 years old).

**Methods:**

A total of 260 patients (265 lesions) with gastric GISTs treated via endoscopic resection from January 2011 to May 2020 were retrospectively analyzed. Among them, 65 patients were ≥65 years old (elderly group), and 195 patients were <65 years old (nonelderly group). Clinicopathological characteristics, postoperative complications, and tumor recurrence rates between the two age groups were compared.

**Results:**

A total of 260 patients with primary small (≤5 cm) gastric GISTs were treated with endoscopic resection. The median ages of the elderly and nonelderly groups were 68 (range 65-83) years and 55 (range 32-64) years, respectively. Elderly patients showed a higher incidence of comorbidities compared with nonelderly patients (61.5% versus 32.3%s, respectively; *p* < 0.001). All elderly patients and 99.0% of nonelderly patients underwent en bloc resection; only two nonelderly patients received piecemeal resection. No significant differences were found regarding postoperative complications or tumor recurrence rates between the two groups.

**Conclusions:**

Although elderly patients had more comorbidities than nonelderly patients, both groups had similar postoperative complications and recurrence rates. We suggest that endoscopic resection performed by experienced endoscopists is safe and effective for treating small (≤5 cm) gastric GISTs in elderly patients.

## 1. Introduction

Gastrointestinal stromal tumors (GISTs) are the most common gastrointestinal tract neoplasm originating from mesenchymal tissue [1], and more than 5000 new cases occur in the United States each year [2]. Most GISTs have gain-of-function mutations in c-KIT or platelet-derived growth factor receptor alpha that promote tumor cell proliferation and inhibit tumor cell apoptosis [3, 4]. Clinical manifestations of GISTs vary greatly from the Carney triad [5] and GIST-paraganglioma syndrome to incidental physical examination findings [6]. Almost all GISTs are considered to have malignant potential, regardless of the size or mitotic index of the tumors [1]. Previous studies have indicated that the stomach is the most frequent location for primary GISTs, followed by the small intestine, colon, rectum, and esophagus [2, 7, 8]. Moreover, gastric GISTs present with a smaller size and fewer symptoms compared with GISTs at other sites [9].

GISTs can appear at any age without significant gender differences [10]. GISTs can be diagnosed at any age, with the peak age of incidence of GISTs ranging from 60 to 74 years [11, 12]. Elderly individuals are more likely to have multiple comorbidities and a worse systematic physical condition compared with younger patients [13], which greatly impact the choice of therapeutic approaches for elderly patients with GISTs. There is a lack of understanding of appropriate therapeutic options for elderly patients with GISTs due to the underrepresentation of clinical trials in this field. With the aging of the global population, further studies are urgently needed regarding treatment strategy exploration for elderly patients with GISTs.

Recently, the increasing availability and universality of endoscopy have significantly augmented the detection rate of GISTs. Open surgery or laparoscopic resection is recommended as the standard treatment for GISTs originating from the muscularis propria [14]. However, the endoscopic treatment of small (≤5 cm) GISTs remains controversial. Some studies have reported that endoscopic resection for GISTs predisposes patients to unavoidable perforation and a positive tumor margin, which results in local recurrence and distant metastasis [15]. Various studies have pointed out that endoscopic resection is a safe and reliable treatment for GISTs [16–18]. Currently, endoscopic resection, including endoscopic submucosal dissection (ESD), endoscopic full-thickness resection (EFTR), and endoscopic submucosal excavation (ESE), has been gradually adopted for treating gastrointestinal tumors [19]. Compared with surgical resection, endoscopic resection has the tremendous advantages of being a less invasive procedure, requiring less administration of sedative or analgesic medications, and having a lower cost and shorter length of postoperative hospital stay [20]. Thus, endoscopic resection seems to be a better therapeutic choice for GISTs.

To date, the clinical outcomes of endoscopic treatment for elderly patients have not been reported. Therefore, it is important to evaluate the efficacy and safety of endoscopic resection in patients with primary gastric GISTs. Here, we conducted a retrospective study that analyzed clinical data from 260 patients with primary small (≤5 cm) gastric GISTs who underwent endoscopic resection between 2011 and 2020 at the First Affiliated Hospital, Zhejiang University School of Medicine (Hangzhou, China), to gain a better understanding of the efficacy and safety of endoscopic resection for small (≤5 cm) gastric GISTs in elderly patients (≥65 years).

## 2. Methods

### 2.1. Patients

In this study, we retrospectively enrolled patients with pathologically confirmed gastric GISTs who underwent endoscopic treatment at the First Affiliated Hospital, Zhejiang University School of Medicine (Hangzhou, China), between January 2011 and May 2020. We received approval from the Research Ethics Committee of the First Affiliated Hospital, Zhejiang University School of Medicine. The inclusion criteria were as follows: (1) the gastric lesions were diagnosed histopathologically as GISTs; (2) the tumors were 5 cm or smaller; and (3) the patients had no evidence of lymph node or distant metastasis. Patients were excluded if they died from a non-tumor-related cause. Finally, 260 patients with gastric GISTs were enrolled. Among them, 65 were aged 65 years or older (elderly group) and 195 were younger than 65 years old (nonelderly group).

### 2.2. Endoscopic Procedures

Before endoscopic resection, patients underwent a preoperative examination to evaluate their physical condition, tumor size, tumor origin, and tumor metastasis. Patients who took anticoagulants or antiplatelets were asked to stop these drugs at least 1 week prior to endoscopic resection if feasible. All the endoscopic procedures were performed by experienced endoscopists. After fasting for 6 h, the patients received endoscopic treatment under general anesthesia with endotracheal intubation. Blood loss was defined as the amount of bleeding during the endoscopic procedure with or without the need for endoscopic hemostasis. After endoscopic resection procedures, all patients underwent gastrointestinal decompression and four-day routine hospital observation. All resected tissues were collected and immediately prepared for pathological examination to determine the diagnosis. Endoscopic resection procedure-related death was defined as death from adverse effects within 30-day postoperation.

### 2.3. Endoscopic Submucosal Dissection

**Figure 1:**
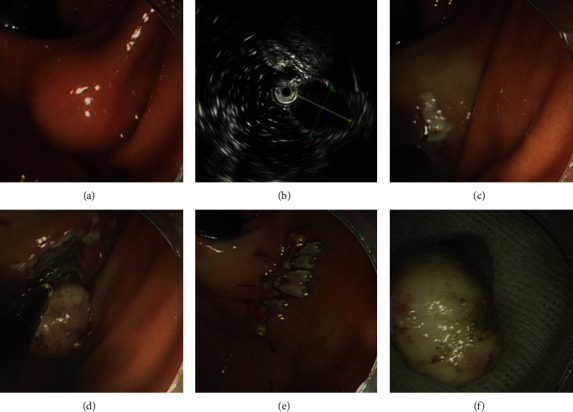
Endoscopic submucosal dissection treatment for a gastric GIST: (a) gastric GIST identified by endoscopy; (b) the same gastric GIST observed by endoscopic ultrasound; (c) marking outside the tumor; (d) dissection of the tumor; (e) close the wound; (f) the resected tumor. GIST: gastrointestinal stromal tumor.

ESD is a common approach used for the resection of GISTs with intraluminal growth patterns. The major steps of ESD are as follows: (1) marking: the electrosurgical knife was used to point the marker dots about 5-10 mm outside the target lesions; (2) injection: the mixed solution (0.9% normal saline solution plus 0.002% indigo carmine plus 0.001% epinephrine) was injected into the submucosal layer to elevate the GISTs; (3) precutting: the needle-knife was used to cut the mucosa along with the marker dots; (4) incision: the muscularis propria layer associated with GISTs was stripped using an insulation-tipped knife; (5) submucosal dissection: the needle-knife was used to completely dissect tumors from the muscularis propria layer; and (6) closing of the wound (Figure 1).

### 2.4. Endoscopic Full-Thickness Resection

**Figure 2:**
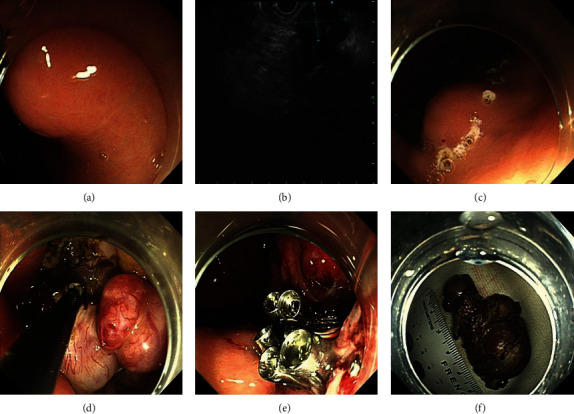
Endoscopic full-thickness resection treatment for a gastric GIST: (a) gastric GIST identified by endoscopy; (b) the same gastric GIST observed by endoscopic ultrasound; (c) marking outside the tumor; (d) resection of the tumor; (e) close the wound; (f) the resected tumor. GIST: gastrointestinal stromal tumor.

EFTR is a common approach used for the resection of GISTs with extraluminal and mixed growth patterns. The first three steps of EFTR are the same as those for ESD. After (1) marking, (2) injection, and (3) precutting, (4) the insulation-tipped knife was used to cut the serosal layer to form a circumferential incision surrounding the tumor. (5) Thereafter, the tumor and surrounding serosal layer were completely removed by snaring. (6) Finally, a loop-and-clip closure technique or over-the-scope clip was used to close the defect (Figure 2).

### 2.5. Endoscopic Submucosal Excavation

**Figure 3:**
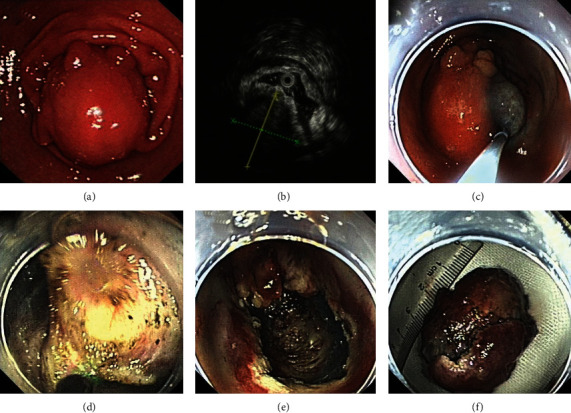
Endoscopic submucosal excavation treatment for a gastric GIST: (a) gastric GIST identified by endoscopy; (b) the same gastric GIST observed by endoscopic ultrasound; (c) marking the tumor; (d) resection of the tumor; (e) the wound after tumor removal; (f) the resected tumor. GIST: gastrointestinal stromal tumor.

ESE is a common approach used for the resection of GISTs with intraluminal growth patterns. The first three steps of EFTR are the same as those for ESD. After (1) marking, (2) injection, and (3) precutting, (4) the insulation-tipped knife or hook knife was used to expose the tumors. (5) Thereafter, the tumor and surrounding tissue were completely resected by snaring. (6) Finally, metallic clips were used to close the defect (Figure 3).

### 2.6. Endoscopic Snare Resection

**Figure 4:**
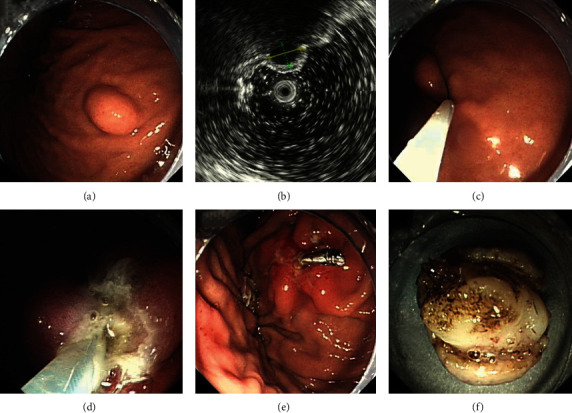
Endoscopic snare resection treatment for a gastric GIST: (a) gastric GIST identified by endoscopy; (b) the same gastric GIST observed by endoscopic ultrasound; (c) encirclement of the tumor by snare; (d) resection of the tumor; (e) close the wound; (f) the resected tumor. GIST: gastrointestinal stromal tumor.

Endoscopic snare resection (ESR) is an approach used for the resection of GISTs with intraluminal growth patterns. After (1) injection, (2) the snare loop was opened to grasp the base of the tumor. (3) Snare resection was conducted by gentle traction and blend current. (4) The tumors were then retrieved using a snare or endoscopic mesh net. (5) Finally, metallic clips were used to close the defect (Figure 4).

#### 2.6.1. Submucosal Tunneling Endoscopic Resection

**Figure 5:**
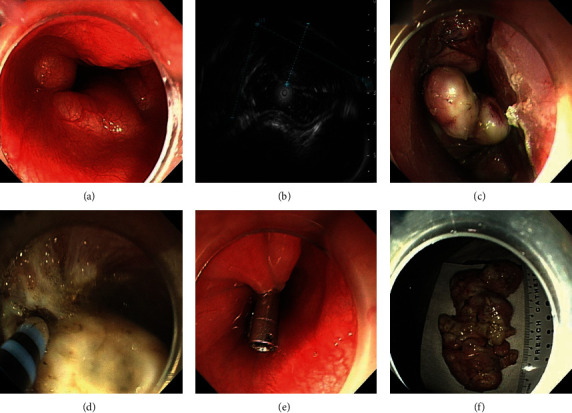
Submucosal tunneling endoscopic resection treatment for a gastric GIST: (a) gastric GIST identified by endoscopy; (b) the same gastric GIST observed by endoscopic ultrasound; (c, d) resection of the tumor via submucosal tunneling; (e) close the wound; (f) the resected tumor. GIST: gastrointestinal stromal tumor.

Submucosal tunneling endoscopic resection (STER) is an approach used for the resection of GISTs with intraluminal growth patterns. After (1) marking and (2) injection, (3) a hook knife was used to cut a 1.5–2.0 cm longitudinal mucosal incision as a tunnel entrance. (4) A submucosal tunnel was created to ensure a sufficient view and workspace. (5) The insulation-tipped knife or hook knife was used for tumor resection. (6) Finally, hemostatic clips were used to close the defect (Figure 5).

### 2.7. Clinicopathologic Variables

Demographic data (age and gender), clinical data (clinical symptoms and comorbidities), tumor characteristics (number of lesions, tumor location, tumor size, tumor growth pattern, and pathological outcome), procedural-related details, and postoperative outcomes were obtained. Based on age, patients were classified into the nonelderly (<65 years old) or elderly (≥65 years old) groups.

### 2.8. Pathology Assessment

The pathology assessment was confirmed by two experienced pathologists. En bloc resection was defined as resection of a tumor in a single piece by endoscopic treatment. Gastric GIST was diagnosed by immunohistochemical staining for CD117 (c-KIT), CD34, DOG-1, S-100, SMA, and desmin on paraffin-embedded specimens. The risk classification of all specimens was based on the modified National Institutes of Health (NIH) risk stratification [21].

### 2.9. Definitions of Postoperative Complications

Delayed bleeding was defined as upper or lower gastrointestinal bleeding requiring an emergency endoscopy hemostatic procedure after endoscopic resection. Perforation was defined as gastric wall penetration diagnosed by the presence of abdominal free air on plain radiography after endoscopic resection.

### 2.10. Follow-Up

Follow-up strategies were conducted via an outpatient service or telephone call. Endoscopic examinations were performed at 1, 3, 6, and 12 months after endoscopic treatment and once yearly thereafter to evaluate the healing of the wound and to exclude local recurrence of the tumor. An abdominal computed tomography scan was performed to check for metastasis every year.

### 2.11. Statistical Analysis

All data analyses were conducted using SPSS statistics version 25.0 software (SPSS Inc., Chicago, IL, USA). Continuous variables were expressed as mean ± standard deviation, and categorical data were expressed as absolute values (*n*) and percentages (%). Continuous variables were compared using the *t*-test or the Mann–Whitney rank sum test, and categorical variables were compared using the chi-squared test or Fisher's exact test. Recurrence-free survival (RFS) was defined as the time from endoscopic resection to diagnosis of tumor recurrence, which was performed using the Kaplan-Meier curve method and log-rank test. All tests were two-sided, and *p* values < 0.05 were considered statistically significant.

## 3. Results

### 3.1. Demographic and Clinicopathological Characteristics

**Figure 6:**
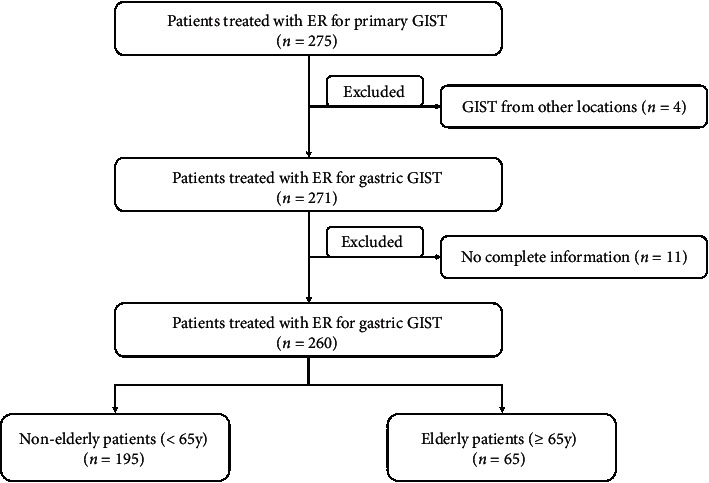
Study flowchart for selection and grouping of patients with gastric gastrointestinal stromal tumors. GIST: gastrointestinal stromal tumor; ER: endoscopic resection.

Of the 275 patients with small (≤5 cm) primary GISTs who underwent endoscopic resection, four patients with GISTs from other locations were excluded and 11 patients were excluded due to incomplete histopathological information (Figure 6). A total of 260 patients with small (≤5 cm) primary gastric GISTs were included in our study, among whom 65 (25%) were aged 65 years or older (elderly group; 65 patients, 66 lesions) and 195 (75%) were younger than 65 years old (nonelderly group; 195 patients, 199 lesions). The demographic and clinical characteristics of the two groups are shown in Tables 1 and 2.

Among these individuals, 116 (44.6%) were male and 144 (55.4%) were female. The median ages of the elderly and nonelderly groups were 68 (range 65-83) years and 55 (range 32-65) years, respectively. Elderly patients showed a higher incidence of comorbidities (elderly group versus nonelderly group; 61.5% versus 32.3%; *p* < 0.001) compared with younger patients. In both groups, the most common comorbidity was hypertension (nonelderly group versus elderly group; 19.0% versus 40.0%; *p* = 0.01), followed by diabetes mellitus (8.7% versus 12.3%; *p* > 0.05), pulmonary disease (6.7% versus 13.8%; *p* > 0.05), and cardiovascular disease (4.6% versus 15.4%; *p* = 0.004). Elderly patients were more susceptible than nonelderly patients to two or more comorbidities (elderly group versus nonelderly group; 20.0% versus 6.7%; *p* = 0.002). There were no significant differences in clinical presentation between the elderly and nonelderly groups. Abdominal pain or discomfort was the most common symptom of gastric GISTs at all ages (39.2%) followed by belching (8.5%), bleeding (3.5%), and dyspepsia (1.5%). Other symptoms including diarrhea, vomiting, wasting, and chest tightness only occurred in nonelderly patients. More than 40% of patients showed no symptoms (nonelderly group, 46.7%; elderly group, 43.1%); therefore, the lesions were found on physical examination. The mean tumor diameters of the nonelderly and elderly groups were 1.75 and 2.04 cm, respectively. Elderly patients exhibited a significantly higher prevalence of ulceration on the tumor surface (nonelderly group versus elderly group; 4.0% versus 12.1%; *p* = 0.017). Only one patient in the elderly group showed an irregular tumor margin. There were no significant differences in the tumor location (the most common location was the gastric fundus), history of alcohol consumption, history of smoking, *Helicobacter pylori* infection, and family history between the elderly and nonelderly groups.

### 3.2. Histopathological Outcome

Tumor tissues were collected for pathological evaluation after endoscopic resection. As presented in Table 2, the cell morphology (spindle, epithelioid, and mixed), tumor growth pattern (intraluminal, extraluminal, and mixed), and mitotic count (≤5/50 high-power field (HPF), 6-10/50 HPF, and >10/50 HPF) were classified into three groups, respectively. For morphology, 97.0% of elderly patients and 97.5% of nonelderly patients presented with a spindle cellular phenotype. For tumor growth pattern, most lesions showed the intraluminal growth pattern (elderly patients, 77.3%; nonelderly patients, 76.9%) in both groups. For mitotic count, most lesions were assessed as ≤5/50 HPF in the elderly (95.5%) and nonelderly (95.0%) groups. According to the modified NIH risk stratification [21], the risk classification of GISTs includes very low risk, low risk, intermediate risk, and high risk. In total, less than 10% of lesions in the elderly (7.6%) and nonelderly (6.5%) groups were high/intermediate risk, and there were no significant differences among them. Immunohistochemically, the positive rate of CD34 (elderly patients, 97.0%; nonelderly patients, 98.0%), CD117 (elderly patients, 98.5%; nonelderly patients, 100.0%), and DOG-1 (elderly patients, 100.0%; nonelderly patients, 98.5%) expressions was more than 95% in both groups.

### 3.3. Perioperative Information and Postoperative Complications

In total, 260 patients (265 lesions) with small (≤5 cm) gastric GISTs received endoscopic resection. ESD was performed in 175 tumors (nonelderly group versus elderly group; 132 patients (133 lesions) versus 41 patients (42 lesions)); EFTR was performed in 53 tumors (39 patients (39 lesions) versus 14 patients (14 lesions)); ESE was performed in 28 tumors (17 patients (19 lesions) versus nine patients (nine lesions)); ESR was performed in five tumors (three patients (four lesions) versus one patients (one lesion)); and STER was performed in only four patients (four lesions) in the nonelderly group. All elderly patients and 99.0% of patients in the nonelderly group underwent en bloc resection, and only two nonelderly patients underwent piecemeal resection. All patients were treated successfully with endoscopic resection.

There were no significant differences in the intraoperative blood loss (nonelderly groups versus elderly groups; 5.96 ± 18.39 ml versus 6.10 ± 25.00 ml; *p* > 0.05), postoperative fasting (3.37 ± 1.18 days versus 3.37 ± 1.43 days; *p* > 0.05), postoperative antibiotic usage (4.62 ± 1.50 days versus 4.61 ± 1.51 days; *p* > 0.05), length of hospital stay (5.93 ± 1.48 days versus 6.05 ± 2.48 days; *p* > 0.05), and hospitalization expenses (25020.83 ± 7093.96 RMB versus 26454.12 ± 7940.84 RMB; *p* > 0.05) between the nonelderly and elderly groups.

The postoperative complications are shown in Table 3. No adverse events were observed in the elderly group. Delayed bleeding occurred in one (0.5%) nonelderly patient, and perforation occurred in another one (0.5%) nonelderly patient; both patients have recovered after endoscopic treatment. No patients died from the ER-related procedure.

### 3.4. Subgroup Analysis Based on the Endoscopic Procedure

**Figure 7:**
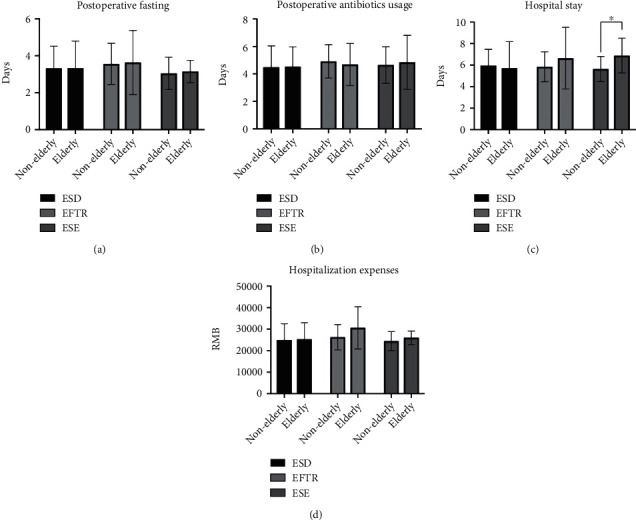
Subgroup analysis on clinical outcomes of elderly and nonelderly patients: (a) postoperative fasting; (b) postoperative antibiotic usage; (c) hospital stay; (d) hospitalization expenses. ESD: endoscopic submucosal dissection; EFTR: endoscopic full-thickness resection; ESE: endoscopic submucosal excavation. ^∗^*p* < 0.05.

Because perioperative complications did not differ significantly, we performed a subgroup analysis based on the endoscopic procedure (Figure 7). Due to the small numbers of patients, subgroup analyses were not performed for ESR and STER. For ESD and EFTR, no significant differences occurred in postoperative fasting, postoperative antibiotic usage, length of hospital stay, or hospitalization expenses between the elderly and nonelderly groups. For ESE, elderly patients had a longer length of hospital stay than nonelderly patients (elderly patients versus nonelderly patients; 6.89 ± 1.62 days versus 5.64 ± 1.17 days, *p* = 0.039). Postoperative fasting, postoperative antibiotic usage, and hospitalization expenses were similar between elderly and nonelderly patients who underwent ESE.

### 3.5. Prognosis of Small (≤5 cm) Gastric GISTs

**Figure 8:**
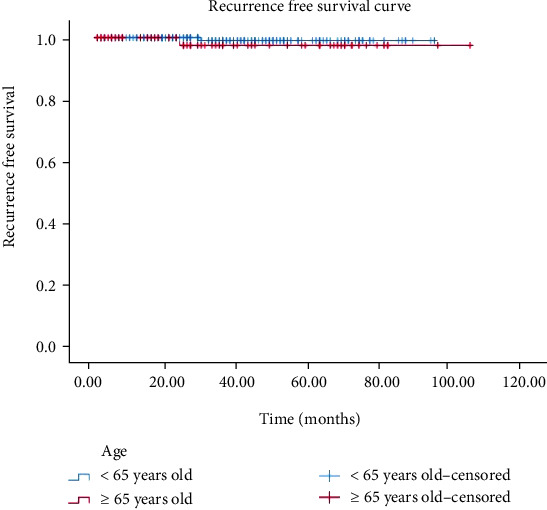
Recurrence-free survival according to age (*p* = 0.395; not significant).

In our study, the median follow-up time was 35 months (range: 1-105 months) for elderly patients and 37 months (range: 2-95 months) for nonelderly patients. In both nonelderly and elderly patients, small (≤5 cm) gastric GISTs showed a favorable prognosis. Only one nonelderly patient and one elderly patient had local tumor recurrence without distant metastases. Detailed clinicopathological features of these two patients are summarized in Table 4. No deaths were observed in either group during the follow-up period. There was no significant difference in the RFS rate between the two groups (*p* = 0.395) (Figure 8).

## 4. Discussion

GISTs are the most common soft tissue sarcomas of the gastrointestinal tract [1], with an annual incidence of 10-15 cases per million [7]. GISTs are characterized by positive expression of CD117 (c-KIT) (95%), CD34 (60-70%), SMA (30-40%), and desmin (1-2%) [22, 23]. Primary GISTs can be found anywhere along the gastrointestinal tract, but the stomach is the most frequent location for these tumors [24]. GISTs may occur anywhere between the ages of 10 and 100 years, while the median age at diagnosis is the sixth decade of life [7]. Previous studies indicated that the risk and incidence of GISTs increased with age [23, 25]. According to the European Medicines Agency, the age of 65 years was defined as the threshold for elderly patients, which has been used in previous studies [26, 27]. Therefore, we also chose 65 years of age as the point at which to divide the patients into different age groups. An article published in the Lancet in 2017 predicted the life expectancy in 35 industrialized countries, indicating that both males and females will have a life expectancy that exceeds 20 years when they are 65 years by 2030 [28]. The rapid growth of the aging population will increase the urgent need for effective treatment of GISTs in elderly patients. Previous studies have indicated that increased attention has been paid to elderly patients during the past decade [29]. Herein, we presented a study that assessed the clinicopathological characteristics of primary gastric GISTs and treatment outcomes of elderly and nonelderly patients to explore the effects of age on the prognosis of primary gastric GISTs and the selection of the therapeutic modality.

Elderly patients with primary gastric GISTs remain a medical challenge, mainly due to the presence of multiple comorbidities and poor physical function. In this study, comorbidities were more frequent in elderly patients than in nonelderly patients. Moreover, elderly patients were more prone to suffer from two or more comorbidities, which is in agreement with previously published literature [12, 30, 31]. We found that gender distributions, tumor location, tumor size, mitotic count, growth pattern, and risk classification are similar between elderly and nonelderly patients, in line with previous findings [32, 33]. The National Comprehensive Cancer Network (NCCN) guidelines state that ulceration is a possible high-risk feature of GISTs [21]. However, no study has investigated the differences in the detection rate for tumor ulceration of primary gastric GISTs between elderly and nonelderly patients. In our study, 12.1% (8/66) of lesions of tumor ulceration occurred in elderly patients, with 4.0% (8/199) in nonelderly patients. Remarkably, despite tumor ulceration being more common in elderly patients than in nonelderly patients, both elderly and nonelderly groups showed the similar clinical outcomes, suggesting that tumor ulceration is not sufficient to evaluate the prognosis of GISTs.

The optimal treatment for small (≤5 cm) GISTs is controversial. The NCCN suggests that very small (<2 cm) gastric GISTs without high-risk EUS features should be monitored periodically and that complete surgical resection should be conducted in patients with high-risk EUS features [21]. The European Society for Medical Oncology suggests that localized GISTs should be completely excised by laparoscopic excision or open surgery [34]. Further, the Japanese Clinical Practice Guidelines for GISTs suggested that small (≤5 cm) GISTs can be excised by endoscopic surgery [35]. Although recent years have seen a development in the endoscopic treatment for small (≤5 cm) GISTs, the increased risk of recurrence caused by perforation and the residual tumor margin in endoscopic procedures has limited the application of endoscopic resection. In fact, many studies have demonstrated that endoscopic resection has several clear advantages for the treatment of small (≤5 cm) GISTs compared to open surgery, including a shorter length of operation time, less intraoperative blood loss, and a lower adverse event rate [20, 36–39]. Moreover, several publications have indicated that minimally invasive approaches, such as ER, laparoscopic surgery, and laparoscopic and endoscopic cooperative surgery, are safe and feasible for elderly patients [40, 41]. In this study, both elderly and nonelderly groups showed similar clinical outcomes. The en bloc resection rates of the two groups were similar, both of which were over 99%. Notably, intraoperative outcomes (intraoperative blood loss), postoperative outcomes (postoperative fasting, postoperative antibiotic usage, and length of hospital stay), and hospitalization expenses were not significantly different between the groups. In our study, the major postoperative complications of endoscopic resection were delayed bleeding and perforation, and no significant difference in the occurrence of postoperative complications was detected between the two age groups, which is similar to the findings of previous studies [31, 42]. Collectively, our results show that endoscopic resection is a safe and economical intervention for both elderly and nonelderly patients. However, it has been reported that age may impact treatment outcomes and there is a trend toward more postoperative complications in elderly patients [43]. Yang et al. indicated that elderly patients had more postoperative complications when compared with nonelderly patients [44]. This phenomenon can be explained by the fact that a high number of elderly patients underwent open surgery in that study. A higher incidence of comorbidities significantly reduces the tolerance of open surgery in elderly patients, which is why postoperative complications are more common in elderly patients who undergo open surgery compared to nonelderly patients. Therefore, endoscopic resection is considered feasible in elderly patients to treat small gastric GISTs.

Studies have shown that age is an important risk factor for predicting gastric GISTs and that elderly patients tend to have a poor prognosis [32, 44, 45]. In contrast, some studies have shown that age is not a sensitive clinical predictor for determining the prognosis for GISTs [26, 46–48], which indicated that the Charlson comorbidity index, prognostic nutritional index, tumor size, and proliferative index could be significant prognostic factors for predicting the prognosis of patients with GISTs. In our study, elderly and nonelderly patients had similar long-term outcomes, and no disease-related deaths were observed during the follow-up period in either group. Moreover, there were no statistically significant differences in RFS between the two age groups. Our findings indicate that treatment decisions for patients with GISTs cannot be determined solely by age.

To the best of our knowledge, this is the first comparative analysis to explore the clinical outcomes of endoscopic treatment for elderly and nonelderly patients with small (≤5 cm) gastric GISTs. However, there are several limitations to our study. The first limitation is the patient selection bias due to the retrospective nature of the study and the small number of elderly patients. Second, the follow-up period of this study was not long enough to explore the long-term outcomes of endoscopic resection. Third, all of the endoscopic procedures were performed by experienced endoscopists, leading to improvement in the of success rates of endoscopic treatment in both groups. Therefore, a large multicenter randomized controlled prospective study with a long follow-up duration is necessary to further verify the long-term outcome of endoscopic treatment in elderly patients with small (≤5 cm) gastric GISTs.

In conclusion, we explored the efficacy and safety of endoscopic resection for small (≤5 cm) gastric GISTs, especially in patients aged 65 years or older. In our study, elderly patients had more comorbidities compared with nonelderly patients. However, the postoperative complications and recurrence rates were similar between elderly and nonelderly patients. Therefore, we suggest that endoscopic resection performed by experienced endoscopists is a safe and effective treatment strategy for small (≤5 cm) gastric GISTs in elderly patients. More studies are needed to further evaluate the long-term outcomes of ER for small (≤5 cm) gastric GISTs in elderly patients.

## Figures and Tables

**Table 1:** Clinical characteristics of the patients.

Characteristics	Nonelderly (<65 years)	Elderly (≥65 years)	*p* value
Number of patients	195	65	
Number of lesions	199	66	
Age, years			<0.001
Median (range)	55 (32-65)	68 (65-83)	
Gender			NS
Male	87 (44.6%)	29 (44.6%)	
Female	108 (55.4%)	36 (55.4%)	
Clinical presentations			
Abdominal pain or discomfort	76 (39.0%)	26 (40.0%)	NS
Belching	15 (7.7%)	7 (10.8%)	NS
Bleeding	6 (3.1%)	3 (4.6%)	NS
Dyspepsia	3 (1.5%)	1 (1.5)	NS
Others^∗^	4 (2.0%)	0 (0.0%)	NS
Asymptomatic	91 (46.7%)	28 (43.1%)	NS
Comorbidity	63 (32.3%)	40 (61.5%)	<0.001
Hypertension	37 (19.0%)	26 (40%)	0.01
Diabetes mellitus	17 (8.7%)	8 (12.3%)	NS
Cardiovascular disease^∗^	9 (4.6%)	10 (15.4)	0.004
Pulmonary disease^∗^	13 (6.7%)	9 (13.8%)	NS
Having two or more complications	13 (6.7%)	13 (20.0%)	0.002
H. pylori infection	19 (9.7%)	2 (3.1%)	NS
History of smoking	46 (23.6%)	15 (23.1%)	NS
History of alcohol consumption	44 (22.6%)	13 (20.0%)	NS
Family history	0 (0.0%)	0 (0.0%)	NS

Data are shown as *n* (%) or mean ± standard deviation of patients. Others^∗^ include diarrhea, vomiting, wasting, and chest tightness. Cardiovascular disease^∗^ includes coronary heart disease, atrial fibrillation, arrhythmia, rheumatic heart disease, and valvular heart disease. Pulmonary disease^∗^ includes pneumonia, chronic bronchitis, bronchiectasia, and pulmonary fibrosis, pulmonary nodules. NS: not significant.

**Table 2:** Clinicopathological characteristics of gastric GISTs.

Characteristics	Nonelderly (<65 years)	Elderly (≥65 years)	*p* value
Number of patients	195	65	
Number of lesions	199	66	
Tumor location			NS
Gastric cardia	6 (3.0%)	3 (4.5%)	
Gastric fundus	98 (49.3%)	32 (48.5%)	
Gastric body	80 (40.2%)	30 (45.5%)	
Gastric antrum	10 (5.0%)	1 (1.5%)	
Anastomosis	5 (2.5%)	0 (0.0%)	
Tumor size			NS
≤2 cm	137 (68.8%)	37 (56.1%)	
2-5 cm	62 (31.2%)	29 (43.9%)	
Tumor ulceration	8 (4.0%)	8 (12.1%)	0.017
Tumor margin			NS
Regular	199 (100.0%)	65 (98.5%)	
Irregular	0 (0.0%)	1 (1.5%)	
Morphology			NS
Spindle	194 (97.5%)	64 (97.0%)	
Epithelioid	1 (0.5%)	0 (0.0%)	
Mixed	4 (2.0%)	2 (3.0%)	
Mitotic count			NS
≤5/50 HPF	189 (95.0%)	63 (95.5%)	
6-10/50 HPF	6 (3.0%)	2 (3.0%)	
>10/50 HPF	4 (2.0%)	1 (1.5%)	
Growth pattern			NS
Intraluminal growth	153 (76.9%)	51 (77.3%)	
Extraluminal growth	34 (17.1%)	13 (19.7%)	
Mixed	12 (6.0%)	2 (3.0%)	
Modified NIH risk categories			NS
Very low risk	85 (42.7%)	20 (30.3%)	
Low risk	101 (50.8%)	41 (62.1%)	
Intermediate risk	9 (4.5%)	4 (6.1%)	
High risk	4 (2.0%)	1 (1.5%)	
IHC			
CD34 (+)	195 (98.0%)	64 (97.0%)	NS
CD117 (+)	199 (100.0%)	65 (98.5%)	NS
DOG-1 (+)	196 (98.5%)	66 (100.0%)	NS

Data are shown as *n* (%) or mean ± standard deviation of patients. NS: not significant; IHC: immunohistochemistry; CD34: CD34 protein; CD117: CD117 protein; DOG1: DOG1 protein; HPF: high-power field; NIH: National Institutes of Health.

**Table 3:** Perioperative information and postoperative complications of patients.

Characteristics	Nonelderly (<65 years)	Elderly (≥65 years)	*p* value
Number of patients	195	65	
Number of lesions	199	66	
Endoscopic method			NS
ESD	133 (66.8%)	42 (63.6%)	
EFTR	39 (19.6%)	14 (21.2%)	
ESE	19 (9.6%)	9 (13.6%)	
ESR	4 (2.0%)	1 (1.5%)	
STER	4 (2.0%)	0 (0.0%)	
Endoscopic procedure			NS
En bloc	197 (99.0%)	66 (100.0%)	
Piecemeal	2 (1.0%)	0 (0.0%)	
Intraoperative blood loss (ml)	5.82 ± 18.30	5.88 ± 24.94	NS
Postoperative fasting (days)	3.37 ± 1.18	3.37 ± 1.43	NS
Postoperative antibiotic usage (days)	4.62 ± 1.50	4.61 ± 1.51	NS
Hospital stay (days)	5.93 ± 1.48	6.05 ± 2.48	NS
Hospitalization expenses (RMB)	25020.83 ± 7093.96	26454.12 ± 7940.84	NS
Postoperative complications			
Delayed bleeding (ml)	1 (0.5%)	0 (0.0%)	NS
Perforation	1 (0.5%)	0 (0.0%)	NS
ER-related death	0 (0.0%)	0 (0.0%)	NS
Recurrence	1 (0.5%)	1 (1.5%)	NS

Data are shown as *n* (%) or mean ± standard deviation of patients. NS: not significant; ESD: endoscopic submucosal dissection; EFTR: endoscopic full-thickness resection; ESE: endoscopic submucosal excavation; ESR: endoscopic snare resection; STER: submucosal tunneling endoscopic resection; ER: endoscopic resection.

**Table 4:** Clinicopathological characteristics of two recurrent patients.

Characteristics	1	2
Age (years)	72	39
Gender	Male	Female
Endoscopic resection	ESE	ESD
Tumor location	Gastric fundus	Gastric body
Tumor size (cm)	2.9	1.5
Mitotic count	>10/50 HFP	<5/50 HFP
Growth pattern	Extraluminal growth	Intraluminal growth
Modified NIH risk categories	High risk	Low risk
Tumor ulceration	Presence	Absence
Tumor margin	Regular	Regular
Endoscopic procedure	En bloc	En bloc
Clinical presentations	Bleeding	Asymptomatic
Comorbidity	Hypertension	Absence
Postoperative complications	Absence	Absence
Recurrence-free time (months)	24	30

ESE: endoscopic submucosal excavation; ESD: endoscopic submucosal dissection; HPF: high-power field; NIH: National Institutes of Health.

## Data Availability

All data of this study could be obtained by emailing the corresponding author.

## Abbreviations

GISTs: Gastrointestinal stromal tumors
ESD: Endoscopic submucosal dissection
EFTR: Endoscopic full-thickness resection
ESE: Endoscopic submucosal excavation
STER: Submucosal tunneling endoscopic resection
NCCN: National Comprehensive Cancer Network
ER: Endoscopic resection.
